# The Omicron variant of concern: The genomics, diagnostics, and clinical characteristics in children

**DOI:** 10.3389/fped.2022.898463

**Published:** 2022-08-02

**Authors:** Djatnika Setiabudi, Yunia Sribudiani, Kartika Hermawan, Basti Andriyoko, Heda Melinda Nataprawira

**Affiliations:** ^1^Division of Infection and Tropical Diseases, Department of Child Health, Faculty of Medicine, Hasan Sadikin General Hospital, Universitas Padjadjaran, Bandung, Indonesia; ^2^Department of Biomedical Sciences, Faculty of Medicine, Universitas Padjadjaran, Bandung, Indonesia; ^3^Division of Respirology, Department of Child Health, Faculty of Medicine, Hasan Sadikin General Hospital, Universitas Padjadjaran, Bandung, Indonesia; ^4^Department of Clinical Pathology, Faculty of Medicine, Hasan Sadikin General Hospital, Universitas Padjadjaran, Bandung, Indonesia

**Keywords:** COVID-19, clinical characteristics, Omicron genomics, Omicron subvariant, SARS-COV2, vaccine

## Abstract

Since WHO announced the COVID-19 pandemic in March 2020, SARS-CoV-2 has undergone several mutations, with the most recent variant first identified in South Africa in November 2021, the SARS-CoV-2 variant of concern (VOC B.1.1.529) named by WHO as Omicron. To date, it has undergone more mutations compared to previous SARS-CoV-2 variants, particularly, in the S gene that encodes the spike protein, which can cause S gene target failure in some PCR kits. Since its discovery, the Omicron variant has caused a sharp rise in COVID-19 cases worldwide and was responsible for a record of 15 million new COVID-19 cases reported globally in a single week, although this may be an underestimate. Since January 2022, Omicron subvariants with variable genetic characteristics, BA.1, BA.1.1, BA.2, BA.3, BA.4, BA.5, and BA.2.12.2 have been identified, with several countries reporting BA.1.1 was the major subvariant (27.42%), followed by BA.2 (25.19%). At the begining of May 2022, BA.2.12.1 mostly (42%) was detected in the United States. Like adults, the clinical manifestations of the Omicron variant in children are similar to the previous variants consisting of fever, cough, vomiting, breathing difficulties, and diarrhea, with some reports on croup-like symptoms and seizures. Though it presents apparently milder disease than the Delta variant, it is significantly more contagious and has caused more hospitalizations, especially in unvaccinated children younger than 5 years and unvaccinated or incompletely vaccinated adults. However, there is insufficient evidence yet to distinguish the Omicron variant from the other variants based solely on the clinical manifestations, therefore, this review presents a brief literature review of the most current evidence and data related to Omicron.

## Introduction

Recently, South Africa reported the identification of a new SARS-CoV-2 VOC Omicron, subvariant BA.1 (also known as B.1.1.529.1), which was first identified from a specimen collected on 9 November 2021 in South Africa (1). This variant has more than thirty mutations in the conserved domain of the spike (S) protein, some of which (e.g., 69–70del, T95I, G142D/143–145del, K417N, T478K, N501Y, N655Y, N679K, and P681H) overlap with those in the Alpha, Beta, Gamma, or Delta variants and nineteen mutations in the conserved non-spike protein (2).

By the end of January 2022, other subvariants BA.2 (B.1.1.529.2) and BA.3 (B.1.1.529.3) had been identified in multiple countries in Europe. The BA.2 subvariant differs by the fifty amino acids from the BA.1 subvariant and substantial differences in the amino acid content, specifically, in the S protein between these two subvariants means the BA.2 may have different virologic characteristics compared with the BA.1. As of January 2022, BA.2 is outcompeting BA.1 in Asia and Europe, suggesting that BA.2 is more contagious due to increased capability of transmission, binding affinity, and the ability to escape the immune system (3–6). This paper presents a brief review of the literature regarding the most current evidence and data related to Omicron (VOC) in terms of the genomic, diagnostic, and clinical characteristics in children.

## Methods

The PubMed and Google Scholar databases were searched for articles and news relevant to Omicron using the following keywords: Omicron subvariant, Omicron vaccination or vaccine, Omicron detection, neutralizing antibodies, and clinical characteristic in children. In total, more than 1,000 articles were identified. In total, sixty articles were manually selected which cover genomics of Omicron, diagnostic, antibodies resistance, vaccine, and clinical characteristics in children.

## Epidemiology

Since it was detected in South Africa in November 2021, the mean number of COVID-19 cases tripled in week (7). The rate of confirmed positive cases increased more rapidly than the previous variant Alpha, Beta, and Delta waves, with Omicron (BA.1.1.529) responsible for the worldwide increase in COVID-19 cases. During the first week of January 2022, more than 15 million new cases of COVID-19 in 149 countries were reported to the WHO, the most in any single week of the pandemic to date, (8, 9) with weekly infections increasing by 65% in Europe, 78% in the Southeast Asia, and 100% in the United States (10). Omicron also became the dominant variant identified in patients with COVID in the Southern African countries such as Botswana, Namibia, Eswatini, Zimbabwe, and Lesotho, and also in Belgium, Israel, and Hong Kong SAR, China (2).

The first United States Omicron case on 1 December 2021 involved six probable cases in one household and the index patient had traveled from Nigeria. He was unvaccinated but had a COVID-19 infection a year prior. All the six household members (median age = 18.5 years; range 11–48 years) tested positive for the SARS-CoV-2 Omicron variant (11).

Of the first forty patients identified in the National Medical Center in South Korea during 4–17 December 2021, 42.5% had traveled recently from countries in Africa (South Africa, Nigeria, and Mozambique) and 2.5% from the United States, with 55% of cases being unvaccinated and 2.5% being partially vaccinated (12).

Based on the rapid increase in Omicron cases in the United States, modeling predicted that the wave would peak by mid-January 2022 and that the number of cases was likely to overwhelm the testing system. The projected mortality was between 60% of the Delta wave, while hospitalization was projected to be comparable to the situation in January 2021 to 4–5 times higher in the most pessimistic scenario (13). On the basis of these observations, the Omicron variant was projected to peak in approximately 32–45 days, (14) therefore, reaching a peak by the end February in Indonesia.

The subvariants of Omicron BA.2 and BA.3 were first identified in the Gauteng Province in South Africa, in the same place as the earlier BA.1 variant. BA.2 was identified at the end of December 2021, spreading rapidly to South Africa, Denmark, and Philippines (15). BA.2 can avoid neutralizing antibodies gained from vaccination or prior infection with BA.1. A Danish study also reported the significantly greater transmission ability of BA.2 from unvaccinated primary cases compared with the BA.1 [odds ratio (OR) = 2.62]. Fortunately, this pattern was not observed in fully vaccinated and booster-vaccinated primary cases. The BA.2 was thought might prolong the Omicron peaks but was unlikely to induce another wave of infection, so would especially be problematic in the areas with lower vaccination rates (16, 17). Based on data from GSAID on 11 May 2022, 3,275,477 Omicron subvariant sequences had been reported from at least 170 countries. Of those subvariants sequences, BA.1.1 was the major subvariant (27.42%), followed by BA.2 (25.19%), and BA.1 (14.46%) (18). In the mid-April 2022, new subvariant BA.2.12.1 was identified in the United States. According to data from Centers for Diseases Control and Prevention (CDC), this subvariant was responsible for 29% of SARS-CoV-2 infection in the mid-April (19). Based on the news from CNBC, on 11 May 2022, subvariant BA.2.12.1 has been detected in 23 countries, furthermore, there were at least 700 cases of BA.4 and more than 300 cases of BA.5 have been detected at least in 16 and 17 countries, respectively. According to United States CDC, BA.4 and BA.5 were estimated to account for 15.7 and 36.6% of new cases, respectively, in the United States by 25 June 2022 (20). The subvariants BA.4 and BA.5 have high rates of detection in South Africa, while BA.2.12.1 mostly was detected in the United States at the beginning of May 2022 (21).

## Omicron genomics

Coronavirus is a non-segmented positive-sense, single-stranded RNA virus (∼32 kb) belonging to the family *Coronaviridae*. Originally, it comprised four genera, *Alphacoronavirus*, *Betacoronavirus*, *Deltacoronavirus*, and *Gammacoronavirus*. It invades the human body using virulence factors consisting of structural proteins, spike (S), membrane (M), nucleocapsid (N), and envelope (E) (22). The S protein contains a receptor-binding domain (RBD) that has an affinity for human angiotensin-converting enzyme 2 (hACE2) which is highly expressed on the surface of epithelial cells in the respiratory tract.

SARS-CoV-2 has evolved and mutated forming several variants through its continuous replication. RNA viruses more easily mutate compared with the DNA viruses, resulting in several changes in the structural proteins causing increased pathogenicity and transmissibility. The WHO classifies new variants of SARS-CoV-2 into four categories according to epidemiology, clinical relevance, pathophysiology, diagnostic and therapeutic effect: variants being monitored (VBM), variant of interest (VOI), VOC, and variant of high consequence (VOHC) (22–25).

Omicron (BA.1, BA.1.1, and BA.2) has 1,270 amino acids, fewer compared to the Delta variant (1,271 amino acids) and the original Wuhan-Hu-1 variant (1,273 amino acids), while BA.3 has 1,276 amino acids (26). Omicron has at least fifty mutations resulting in 23–32 amino acid substitutions, deletions, and insertions, with more than thirty mutations involving the S protein (Table 1). The multiple alignment of all the Omicron subvariants BA.1, BA.1.1, BA.2, and BA.3 with the wild type (Wuhan-1) showed that they have 39, 40, 31, and 34 mutations, respectively, with 21 shared mutations (26). Half of S gene mutations are in the RBD, which is the primary target of monoclonal antibody-based therapy (27). In total, twenty-six mutations are a combination of Delta and Beta variant mutations (Table 1). BA.1.1 has one unique mutation which is R346K, BA.2 has eight unique mutations T19I, L24del (deletion), P25del, P26del, A27S, V213G, T376A, and R408S and BA.3 has one unique mutation R216del. All the subvariants share eleven common mutations (G339D, S373P, S375F, K417N, N440K, S477N, T478K, E484A, Q493R, Q498R, and N501Y) in the RBD of the S protein, which are responsible for enhanced transmission, evasion, antibody neutralization, and RNA expression (26).

**TABLE 1:** Spike gene mutations in the beta, delta, and Omicron variants.

Variant (Subvariant)	Sequence ID	Mutation*
Wuhan-1 (wild type)	NCBI ID- MN_908947.3	–
Alpha (B.1.1.7)	–	Δ69-70, Δ144, **N501Y**, A570D, D614G, P681H, T716I, S982A, D1118H (61)
Beta (B.1.351)	–	D80A, D215G, ΔL242-L244, R264I, **K417N, E484K, N501Y**, D614G, A701V (61–63).
Delta (B.1.617.2)	NCBI: QWK65230.1	T19R, G142D, Δ156-157, R158G, Δ213-214, **L452R, T478K**, D614G, P681R, D950N (61).
Omicron (B.1.1.529)	GSAID ID: R40B60_BHP_ 3321001247/2021	A67V, Δ69-70, T95I, G142D, Δ143-145, N211I, L212V, ins213-214RE, V215P, R216E, **G339D, S371L, S373P, S3735F**, **K417N**, **N440K, G446S, S477N**, **T478K**, **E484A, Q493R**, **G496S, Q498R, N501Y**, **Y505H**, T547K, D614G, H655Y, N679K, P681H, N764K, D796Y, N856K, Q954H, N969K, L981F (61).

*Bold letters indicate mutations located in the RBD.

All the subvariants have more positively charged amino acids compared with the wild type, which may improve their tendency to bind negatively charged proteins such as hACE2 (24, 28–30). Kumar et al. (30) showed that Omicron and its subvariants have a higher affinity for human angiotensin-converting enzyme 2 (ACE2) compared with the wild type and Delta variants because of a significant number of mutations in the RBD, therefore, a potentially higher transmission rate (30). Computational analysis perform by Gan et al. (31) showed that Omicron Spike trimer surface has transitioned to strongly positive electrostatic surface compared to Wuhan-Hu-1 variant. This result suggest that Omicron mutations not only enhance ACE2 recognition but also cause antibody escape (31). BA.2 and BA.3 have a higher transmission potential compared with the BA.1 and BA.1.1 (26). According to a study in the Danish population which showed BA.2 is associated with increased susceptibility to infection with an OR in unvaccinated, fully vaccinated, and booster-vaccinated individuals of 2.19 (95% CI: 1.58–3.04), 2.45 (95% CI: 1.77–3.40), and 2.99 (95% CI: 2.11–22 4.24), respectively, compared with the BA.1. Furthermore, the estimated secondary rate attack (SRA) for BA.2 was higher (39%) than BA.1 (29%) (17). Contact tracing study in England reported that secondary attack rates is higher among contacts of BA.2 subvariants (13.4%; 95% CI: 10.7–16.8%) compared with other Omicron subvariants (10.3%; 95% CI: 10.1–10.4%) in the period of December 2021 to January 2022 (32).

Omicron BA.1 and BA.1.1 subvariants show negative or significantly weaker positive S gene results, with positive results for the other gene targets in some multiplex RT–PCR assays because of the mutations in the spike gene such as His69_Val70del (Δ69-70). Nonetheless, these kits can still detect SARS-CoV-2 because there are other target genes not impacted by mutation. The phenomenon is called the S Gene Target Failure (SGTF) and has been used to identify particular variants. Interpretation of SGTF should be done in cycle threshold 30 or less for other genes to avoid misinterpretation for S gene not detected because of low-viral load. Omicron subvariants comparison study performed by Latif et al. (18) showed that only less than 0.1% of BA.2 and 85.4% of BA.3 have Δ69–70 mutation (18). Thus, most of BA.2 and 14.5% of BA.3 subvariants will give positive signal when using SGTF PCR kit. This phenomenon is called S Gene Positive Test (SGPT) (32, 33). RT–PCR kits that detect this specific mutation on S gene are used as a proxy test for Omicron pending-sequencing confirmation, (33) allowing rapid tracking of Omicron transmission compared with the other variants (34). Commercial multiplex RT–PCR or RT–PCR melting curve analysis for genotyping SARS-CoV-22 variants using single nucleotide polymorphisms (SNPs) panel assays have been developed to differentiate the Alpha, Beta, Gamma, Delta, and Omicron (VOC/VOI) variants (Table 2). However, this kit is not intended for the diagnosis, rather it is a rapid method to estimate the prevalence of specific mutation-positive variants in the population when access to whole genome sequencing (WGS) facilities is limited (33).

**TABLE 2:** The combination of mutations that can be used as a proxy marker to identity SARS-CoV-2 variants (33).

Spike amino acid variation	Alpha B.1.1.7	Beta B.1.351	Gamma B.1.1.28	Delta B.1.6.17	Omicron B.1.15.29
Δ69–70	√				√* BA.1
ins214EPE					x
S371L/S373P					√* BA.1
L452R				√	
N501Y	√	√	√		√
K417T			√		
K417N		√			(√)
E484K		√	√		
E484Q	(√)				
E484A					√
P681H	√				√
P681R				√	
T478K				√	

(√): Some of the VOC sequences carry this amino acid substitution.

*Some of the amino acid variations are different for BA.1 and BA.2 (e.g., Δ69–70 will identify mostly in BA.1 viruses).

## Antibody resistance and vaccination

As more than half of the mutations in Omicron are located in S gene, this rise a concern of their impact on vaccine infectivity and the antibody resistance. This due to the fact that antibodies which binds strongly to RBD has been proven would directly neutralized the virus (35). Study by Takashita et al. (36) showed that among 7 monoclonal antibody tested in Omicron variant (B.1.1.529), Etesevimab, Bamlanivimab, and Imdevimab did not neutralized Omicron even with FRNT_50_ (Focus Reduction Neutralizing Assay 50) >50,000 (27). Casirivimab, Tixagevimab, Cilgavimab, and Sotrovimab retained neutralizing activity against Omicron with a higher FRNT_50_ value than for Beta or Gamma. Furthermore, the combination of Tixagevimab–Cilgavimab could inhibit Omicron with the FRTN_50_ higher by the factor 24.8 and 142.9 than for Beta and Gamma, respectively (27). By using a magnetic-activated cell sorting (MACS)-based screening to characterize the profile of RBD escaping mutations for total 247 neutralizing antibodies, Cao et al. (37) showed that 85% of the tested antibodies were escaped by Omicron (37). While BA.2 subvariant is almost completely resistant to the therapeutic monoclonal antibodies such as Casirivimab and Imdevimab (6).

The fully and partially vaccinated individuals are highly protected from hospitalization related to Delta and Alpha infection. However, some studies showed that several vaccines reduce their effectiveness in Omicron infection (38). Study by Yamasoba et al. (6) showed that BA.2, like BA.1, is highly resistant to vaccine-induced antisera, and also the convalescent sera of those previously infected by early pandemic SARS-CoV2 (before May 2020) (6). Study by the largest private health insurer in South Africa showed that the vaccine effectiveness against Omicron infection reduce to 33% from 80% for Delta. While the Pfizer-BioNtech vaccine’s efficacy againts severe disease and hospitalization reduce to 70% from 93% (39). Previous study during delta variant (B.1.617.2) wave showed that although the vaccine efficacy reduce against new emerging variant infection, however, the protection against severe COVID-19 infection and hospitalization remains high. Furthermore, administration of the booster dose of mRNA-vaccine at least 5–6 month after completion of initial vaccine doses is significantly reducing the risk of infection (40). This situation is similar to Omicron wave, in which vaccine booster could provide higher protection. Initial laboratory study was release by Pfizer and Biontech on 8 December 2021 demonstrating that “serum antibodies induced by the Pfizer-BioNTech COVID-19 Vaccine (BNT162b2) neutralize the SARS-CoV-2 Omicron variant after three doses” [biontech, (41)]. Neutralization of Omicron was not detectable in most mRNA-based vaccine, however, individual boosted with mRNA-based vaccine showed potent neutralization of Omicron by 4–6-fold lower than wild type (38). UK Health Security Agency (UKSHA) reported that COVID-19 vaccine booster can provide higher protection against Omicron and reduces risk of hospitalization up to 88% in the infected patients (42).

## Clinical characteristic of Omicron infection in children

Omicron is different compared with the other SARS-CoV-2 variants, for instance, SARS-CoV-2 mainly manifests in the lower respiratory tract while Omicron affects the upper respiratory tract (42). Experiments performed using the lung organoids showed that many lung cells protrude the TMPRSS2 surface protein, a cofactor of SARS-CoV-2 entry, while this protein is notably absent in most cells of the upper respiratory system. The S protein of Omicron is not well recognized by TMPRSS2 unlike other SAR-CoV-2 variants, which might reduce S protein priming by TMPRSS2 and the internalization of Omicron into the cells (43). This may account for why Omicron is presented in a much lower concentration in the lungs of rodents infected by Omicron compared with those in the upper respiratory tract (44, 45). This data is corroborated with the *in vitro* study performed by Peacock, et al. (46) which showed that Omicron is less reliant on TMPRSS2 for cell entry compared to the previous identified SARS-CoV-2 variants. Furthermore, Omicron replicates rapidly in the nasal epithelium and bronchus (46). An *ex vivo* study by Chan et al. reported that 24 h after infection, Omicron replicates seventy times faster than Delta in the human bronchus but more than ten times slower than Delta and the original SARS-CoV-2 virus in the human lung tissues, (47) explaining the increased transmissibility of Omicron but with milder disease severity. Unlike other variants, Omicron can enter the cell without binding to the ACE2 receptor and activate TMPRSS2, consequently having the ability to invade a wider variety of cells (46, 47). Compared to other variants, Omicron also has the shortest incubation period (3 vs. 4 days for Delta and ≥5 days for other variants) (11).

Currently, children are reported to have an increased hospitalization rate but the cases are mostly mild. Regarding previous variants, review cases from the Chinese Center of Disease Control and Prevention reported that less than 1% of the cases were in children younger than 10 years of age (48). In contrast to infected adults, most pediatric COVID-19 cases have milder symptoms, reported in the international literature as fever, cough, rhinorrhea, diarrhea, nasal symptoms, nausea/vomiting, or no symptoms (49, 50). Until now, only a few studies have reported the clinical features of Omicron infection in children. The multicenter observational study from South Africa identified various clinical manifestations in 138 children infected with Omicron with the most prevalent symptoms being fever (61%), cough (57%), vomiting (26%), shortness of breath (31%), seizures (31%) and diarrhea (25%), (51) with standard care provided to 88% of hospitalized children, 20% required oxygen therapy, 5% were ventilated, and 3% died during the study period. All the deceased cases were related to complex underlying co-pathologies. The duration of hospitalization was also short (mean 3.2 days) and the mean age of hospitalized children was 4.2 years with infants (35%) as the most predominant age group admitted. The case fatality rate was 2.2% which is lower than in the pre-Omicron period (3.6%), with no child dying primarily due to the COVID-19 infection (51).

Wang et al. (52) assessed the severity of Omicron in children less than 5 years old in the United States compared with the Delta variant, showing that children infected with Omicron had a significantly lower risk of severe clinical outcomes including emergency department visits, hospitalizations, ICU admissions, and mechanical ventilation within 3 days of infection (52). Similar trends were observed for other pediatric age groups 5–11 and 12–17 years (53). However, because of the greater transmissibility, the total number of hospitalizations might still be larger in children infected by the Omicron variant. As the data from the United Kingdom showed, there was an increase in hospital admissions for children younger than 1 year, who accounted for 42.2% of children hospitalized from mid-December 2021 to mid-January 2022 (54). Nonetheless, the children admitted to hospital with Omicron had less severe manifestations, required less support, and were discharged earlier than children admitted in the previous waves. This phenomenon was deemed normal in the winter months when many other respiratory viruses were also circulating causing numerous infants to present with high fever and respiratory distress (54). Similar to Wang et al. (52), a recent study by Clark et al. (55) also showed that infants infected with Omicron were predominantly <3 months old and of fifty-five confirmed Omicron cases, forty-five were hospitalized with an average of 2 days. The main reason for admission was fever and/or respiratory symptoms but less than 10% required supplemental oxygen (55).

There have been several reports that children infected with Omicron present with croup-like symptoms consisting of barky cough, breathing difficulties, and occasional fever. These children often need hospital admission while croup can be managed in the emergency department. Children presenting with croup during the Omicron period were significantly more likely to test positive for COVID-19 compared with the Delta period (56). Murata et al. (57) reported that Omicron-induced croup cases were less responsive to the nebulized epinephrine and corticosteroids, requiring longer for improvement (56, 57). Further studies are necessary to determine whether croup-like symptoms are unique to Omicron but this is consistent with Omicron causing severe inflammation in the upper respiratory tract in comparison to the other variants that cause inflammation in the lower respiratory tract.

A study in Sweden also reported that seizures might be a unique feature of Omicron infection in children (58). Similar to a report from South Africa, the seizures occurred in children outside the typical age range for febrile seizures, so the mechanism of traditional febrile seizures cannot explain this phenomenon (58). A study in Wuhan reported that 36% of adult hospitalized patients showed neurological condition associated with COVID-19 (59). Although it is rare, but there are several reports of the neurologic involvement in children developing multisystem inflammatory syndrome in children (MIS–C). This condition appears in the last 2–6 weeks of SARS-CoV2 infection. Among those with neurologic involvement, 12% developing life-threatening condition including severe anchelpetty, stroke, and central nervous infection/demyelination (59). Children with MIS–C oftenly admitted to pediatric intensive unit as they are mostly in critical condition. In the early report of SARS-COV2 infection, MIS–C had been suggested predominantly occured in children older than 5 years and adolescent. However, to date there are not so many reports about MIS–C during Omicron wave. Multicenter observational study in Tshwane–South Africa showed that from 183 hospitalized children, none of them develop MIS–C (51).

Although many data showed that Omicron seems to cause less severe infection as compared to delta, however, we should be aware that the Omicron pandemic is likely multifaceted. Mathematical-based modeling study in Korea showed that non-pharmaceutical intervention (such as social distancing level), vaccination and antiviral therapy influence the spread of Omicron and number of severe cases (60).

## Conclusion

The Omicron appears to be less severe but more contagious than the previous SARS-CoV-2 variants, with symptoms tending to manifest in the upper respiratory tract. Omicron could escape neutralizing antibodies and weaken existing COVID-19 vaccines protection, but vaccine boosters could improve immunity. There is insufficient information to suggest that Omicron causes different manifestations from other variants, however, there are increasing cases of children presenting with croup-like symptoms and seizures. Determination of Omicron VOC is important for surveillance but unlikely to impact the clinical management of COVID-19 cases. Furthermore, due to limited access to WGS globally to confirm Omicron, the PCR-based method using primers specifically recognizing specific mutation(s) in certain variants, such as the SGFT kit, is useful for the rapid genomic surveillance.

## Author contributions

DS, YS, and HN developed the structure of the manuscript. DS, YS, KH, and HN were the major writers of the manuscript. YS and BA wrote the diagnostic part of this manuscript. All authors reviewed the manuscript.

## References

[1] World Health Organization [WHO]. *Classification of Omicron (B.1.1.529): SARS-CoV-2 Variant of Concern.* Geneva: World Health Organization (2021).

[2] Rao S Singh M. The newly detected B.1.1.529 (Omicron) Variant of SARS-CoV-2 With Multiple Mutations Implications for transmission, diagnostics, therapeutics, and immune evasion. *DHR Proc.* (2021) 1:7–10. 10.47488/dhrp.v1iS5.35

[3] Karim SSA Karim QA. Omicron SARS-CoV-2 variant: a new chapter in the COVID-19 pandemic. *Lancet.* (2021) 398:2126–8. 10.1016/S0140-6736(21)02758-6 34871545 PMC8640673

[4] Greaney AJ Starr TN Gilchuk P Zost SJ Binshtein E Loes AN Complete mapping of mutations to the SARS-CoV-2 spike receptor-binding domain that escape antibody recognition. *Cell Host Microbe.* (2021) 29:44–57.e9. 10.1016/j.chom.2020.11.007 33259788 PMC7676316

[5] Harvey WT Carabelli AM Jackson B Gupta RK Thomson EC Harrison EM SARS-CoV-2 variants, spike mutations and immune escape. *Nat Rev Microbiol.* (2021) 19:409–24. 10.1038/s41579-021-00573-0 34075212 PMC8167834

[6] Yamasoba D Kimura I Nasser H Morioka Y Nao N Ito J Virological characteristics of SARS-CoV-2 BA.2 variant. *bioRxiv.* [Preprint]. (2022). 10.1101/2022.02.14.480335

[7] Department of Health. *Government of South Africa COVID-19.* (2022). Available online at: https://sacoronavirus.co.za/ (Accessed January 15, 2022).

[8] World Health Organization [WHO]. *WHO Director-General’s Opening Remarks at the Media Briefing On COVID-19 - 12 January 2022.* (2022). Available online at: https://www.who.int/director-general/speeches/detail/who-director-general-s-opening-remarks-at-the-media-briefing-on-covid-19—12-january-2022 (Accessed January 16, 2022).

[9] World Health Organization [WHO]. *Enhancing response to Omicron SARS-CoV-2 variant: Technical Brief and Priority Actions for Member States.* (2022). Available online at: https://www.who.int/publications/m/item/enhancing-readiness-for-omicron-(b.1.1.529)-technical-brief-and-priority-actions-for-member-states (Accessed March 9, 2022).

[10] Taylor L. Covid-19: omicron drives weekly record high in global infections. *BMJ.* (2022) 376:o66. 10.1136/bmj.o66 35017144

[11] Jansen L Tegomoh B Lange K Showalter K Figliomeni J Abdalhamid B Investigation of a SARS-CoV-2 B.1.1.529 (Omicron) variant cluster — Nebraska, November–December 2021. *Morb Mortal Wkly Rep.* (2021) 70:1782–4. 10.15585/mmwr.mm705152e3 34968376 PMC8736273

[12] Kim MK Lee B Choi YY Um J Lee KS Sung HK Clinical Characteristics of 40 Patients Infected With the SARS-CoV-2 Omicron Variant in Korea. *J Korean Med Sci.* (2022) 37:6–10. 10.3346/jkms.2022.37.e31 35040299 PMC8763884

[13] Covid19-Scenario-Modeling-Hub. *Covid 19 Scenario Modeling Hub.* (2022). Available from: https://covid19scenariomodelinghub.org/viz.html (Accessed March 5, 2022).

[14] Schlickeiser R Kr M. Forecast of omicron wave time evolution. *COVID.* (2022) 2:216–29. 10.3390/covid2030017

[15] Viana R Moyo S Amoako DG Tegally H Scheepers C Althaus CL Rapid epidemic expansion of the SARS-CoV-2 Omicron variant in southern Africa. *Nature.* (2022) 603:679–86. 35042229 10.1038/s41586-022-04411-y PMC8942855

[16] Callaway E. Omicron sub-variant: what scientists know so far. *Nature.* (2022) 602:556–7. 10.1038/d41586-022-00471-2 35177843

[17] Lyngse FP Kirkeby CT Denwood M Christiansen LE Mølbak K Møller CH Transmission of SARS-CoV-2 Omicron VOC subvariants BA.1 and BA.2: evidence from Danish Households. *medRxiv.* [Preprint]. (2022). 10.1101/2022.01.28.22270044

[18] Latif AA Mullen JL Alkuzweny M Tsueng G Cano M Haag E *Omicron Variant Report.* (2022). Available online at: https://outbreak.info/situation-reports/omicron (Accessed March 10, 2022).

[19] Smith-Schoenwalder C. *New Omicron Subvariant Spreading in U.S. as Coronavirus Cases Increase.* (2022). Available online at: https://www.usnews.com/news/national-news/articles/2022-05-02/new-omicron-subvariant-ba-2-12-1-spreading-in-u-s-as-coronavirus-cases-increase (Accessed May 15, 2022).

[20] Centers for Disease Control and Prevention [CDC]. *COVID data Tracker.* (2022). Available online at: https://covid.cdc.gov/covid-data-tracker/#variant-proportions (Accessed June 29, 2022).

[21] Constantino AK. *WHO says omicron BA.4 and BA.5 subvariants have spread to over a dozen countries.* (2022). Available online at: https://www.cnbc.com/2022/05/11/who-says-omicron-bapoint4-and-bapoint5-subvariants-have-spread-to-over-a-dozen-countries.html (Accessed May 15, 2022).

[22] Zeyaullah M AlShahrani AM Muzammil K Ahmad I Alam S Khan WH COVID-19 and SARS-CoV-2 variants: current challenges and health concern. *Front Genet.* (2021) 12:693916. 10.3389/fgene.2021.693916 34211506 PMC8239414

[23] Vasireddy D Vanaparthy R Mohan G Malayala SV Atluri P. Review of COVID-19 Variants and COVID-19 vaccine efficacy: what the clinician should know?. *J Clin Med Res.* (2021) 13:317–25. 10.14740/jocmr4518 34267839 PMC8256910

[24] Ingraham NE Ingbar DH. The omicron variant of SARS-CoV-2: understanding the known and living with unknowns. *Clin Transl Med.* (2021) 11:e685. 10.1002/ctm2.685 34911167 PMC8673343

[25] Center for Disease Control and Prevention [CDC]. *SARS-CoV-2 Variant Classifications and Definitions.* (2022). Available online at: https://www.cdc.gov/coronavirus/2019-ncov/variants/variant-classifications.html (Accessed January 22, 2022).

[26] Kumar S Karuppanan K Subramaniam G. Omicron (BA.1) and sub-variants (BA.1, BA.2 and BA.3) of SARS-CoV-2 spike infectivity and pathogenicity: a comparative sequence and structural-based computational assessment. *bioRxiv.* [Preprint]. (2022). 10.1101/2022.02.11.480029 PMC9347785 35680610

[27] Olson SM Newhams MM Halasa NB Price AM Boom JA Sahni LC Effectiveness of BNT162b2 Vaccine against Critical Covid-19 in Adolescents. *N Engl J Med.* (2022) 386:713–23. 10.1056/NEJMoa2117995 35021004 PMC8781318

[28] Thakur V Ratho RKOMICRON. (B.1.1.529): a new SARS-CoV-2 variant of concern mounting worldwide fear. *J Med Virol.* (2021) 94:1821–4. 10.1002/jmv.27541 34936120

[29] Qin S Cui M Sun S Zhou J Du Z Cui Y Genome characterization and potential risk assessment of the novel SARS-CoV-2 variant omicron (B.1.1.529). *Zoonoses.* (2021) 1:1–5. 10.15212/ZOONOSES-2021-0024

[30] Kumar S Thambiraja TS Karuppanan K Subramaniam G. Omicron and delta variant of SARS-CoV-2: a comparative computational study of spike protein. *J Med Virol.* (2022) 94:1641–9. 10.1002/jmv.27526 34914115

[31] Gan HH Zinno J Piano F Gunsalus KC. Omicron Spike protein has a positive electrostatic surface that promotes ACE2 recognition and antibody escape. *bioRxiv.* [Preprint]. (2022). 10.1101/2022.02.13.480261

[32] UK Health Security Agency. *SARS-CoV-2 variants of concern and variants under investigation in England.* Thousand Oaks: SAGE Publications (2021).

[33] World Health Organization [WHO]. *Methods for the Detection and Identification of SARS-CoV-2 Variants Summary Diagnostic screening Assays of Known VOCs S-Gene Drop Out Or Target Failure.* Geneva: World Health Organization (2021).

[34] Li A Maier A Carter M Guan TH. Omicron and S-gene target failure cases in the highest COVID-19 case rate region in Canada—December 2021. *J Med Virol.* (2021) 94:1784–6. 10.1002/jmv.27562 34964500 PMC9015418

[35] Wang C Li W Drabek D Okba NMA van Haperen R Osterhaus ADME A human monoclonal antibody blocking SARS-CoV-2 infection. *Nat Commun.* (2020) 11:2251. 10.1038/s41467-020-16256-y 32366817 PMC7198537

[36] Takashita E Kinoshita N Seiya Y Fujisaki S Ito M Iwatsuki-Horimoto K Efficacy of antiviral agents against the SARS-COV-2 omicron subvariant BA.2. *N Engl J Med.* (2022) 386:1475-7. 10.1056/NEJMc2201933 35263535 PMC8929374

[37] Cao Y Wang J Jian F Xiao T Song W Yisimayi A Omicron escapes the majority of existing SARS-CoV-2 neutralizing antibodies. *Nature.* (2022) 602:657–63. 10.1038/s41586-021-04385-3 35016194 PMC8866119

[38] Garcia-Beltran WF St. Denis KJ Hoelzemer A Lam EC Nitido AD Sheehan ML mRNA-based COVID-19 vaccine boosters induce neutralizing immunity against SARS-CoV-2 Omicron variant. *Cell.* (2022) 185:457–66.e4. 10.1016/j.cell.2021.12.033 34995482 PMC8733787

[39] Ren S-Y Wang W-B Gao R-D Zhou A-M. Omicron variant (B.1.1.529) of SARS-CoV-2: mutation, infectivity, transmission, and vaccine resistance. *World J Clin Cases.* (2022) 10:1–11. 10.12998/wjcc.v10.i1.1 35071500 PMC8727245

[40] Mohamed K Rzymski P Islam MS Makuku R Mushtaq A Khan A COVID-19 vaccinations: the unknowns, challenges, and hopes. *J Med Virol.* (2022) 94:1336–49. 10.1002/jmv.27487 34845731 PMC9015467

[41] Biontech P *Pfizer and BioNTech Provide Update on Omicron Variant*. (2022). Available online at: https://www.pfizer.com/news/press-release/press-release-detail/pfizer-and-biontech-provide-update-omicron-variant (accessed June 27, 2022)

[42] Khan A Bibi S Kanwal H Umm-e-Kalsoom Hussain H. Omicron: a new face of COVID-19 pandemic. *Heal Sci Rep.* (2022) 5:2021–2. 10.1002/hsr2.526 35224223 PMC8855491

[43] Meng B Ferreira IAT Abdullahi A Saito A Kimura I Yamasoba D SARS-CoV-2 Omicron spike mediated immune escape, infectivity and cell-cell fusion. *bioRxiv.* [Preprint]. (2021). 10.1101/2021.12.17.473248

[44] Diamond M Halfmann P Maemura T Iwatsuki-Horimoto K Iida S Kiso M The SARS-CoV-2 B.1.1.529 Omicron virus causes attenuated infection and disease in mice and hamsters. *Res Sq.* [Preprint]. (2021). 10.21203/rs.3.rs-1211792/v1 34981044 PMC8722607

[45] Kozlov M. Omicron makes a feeble attack on the lungs. *Nature.* (2022) 601:177–177. 10.1038/d41586-022-00007-8 34987210

[46] Peacock TP Brown JC Zhou J Thakur N Newman J Kugathasan R The SARS-CoV-2 variant, Omicron, shows rapid replication in human primary nasal epithelial cultures and efficiently uses the endosomal route of entry. *bioRxiv.* [Preprint]. (2022). 10.1101/2021.12.31.474653

[47] Chi-wai M. HKU Med Finds Omicron SARS-CoV-2 Can Infect Faster and Better than Delta in Human Bronchus but With Less Severe Infection in Lung. (2016).

[48] Zhao Q Meng M Kumar R Wu Y Huang J Lian N Correspondence SARS-CoV-2 Infection in Children. *N Engl J Med.* (2020) 382:1663–1665. 10.1056/NEJMc2005073 32187458 PMC7121177

[49] de Souza TH Nadal JA Nogueira RJN Pereira RM Brandão MB. Clinical manifestations of children with COVID-19: a systematic review. *Pediatr Pulmonol.* (2020) 55:1892–9. 10.1002/ppul.24885 32492251 PMC7300659

[50] Yasuhara J Kuno T Takagi H Sumitomo N. Clinical characteristics of COVID-19 in children: a systematic review. *Pediatr Pulmonol.* (2020) 55:2565–75. 10.1002/ppul.24991 32725955

[51] Cloete J Kruger A Masha M du Plessis NM Mawela D Tshukudu M Paediatric hospitalisations due to COVID-19 during the first SARS-CoV-2 omicron (B.1.1.529) variant wave in South Africa: a multicentre observational study. *Lancet Child Adolesc Heal.* (2022) 6:294–302. 10.1016/S2352-4642(22)00027-X PMC8856663 35189083

[52] Wang L Berger NA Kaelber CD Davis PB Volkow ND Xu R. COVID infection severity in children under 5 years old before and after Omicron emergence in the US. *medRxiv.* [Preprint]. (2022). 10.1101/2022.01.12.22269179 35043116 PMC8764724

[53] Wang L Berger NA Kaelber DC Davis PB Volkow ND Xu R. Comparison of outcomes from COVID infection in pediatric and adult patients before and after the emergence of Omicron. *medRxiv.* [Preprint]. (2022). 10.1101/2021.12.30.21268495 35018384 PMC8750707

[54] Torjesen I. Covid-19: omicron variant is linked to steep rise in hospital admissions of very young children. *BMJ.* (2022) 376:o110. 10.1136/bmj.o110 35031537

[55] Clark M Walker B Bennett E Herrick A Kenny S Gent N. Clinical characteristics of SARS-CoV-2 Omicron infection in children under one year. *SSRN Electron J.* [Preprint]. (2022). 10.2139/ssrn.4013461

[56] Tunç EM Shin CKJ Usoro E Thomas-Smith SE Trehan I Migita RT Croup during the COVID-19 omicron variant surge. *medRxiv.* [Preprint]. (2022). [preprint], 10.1101/2022.02.02.22270222 PMC9085454 35551925

[57] Murata Y Tomari K Matsuoka T. Children with croup and SARS-CoV-2 infection during the large outbreak of omicron. *Pediatr Infect Dis J.* (2022) 41:e249. 10.1097/INF.0000000000003484 35185142 PMC8997017

[58] Ludvigsson JF. Convulsions in children with COVID-19 during the Omicron wave. *Acta Paediatr Int J Paediatr.* (2022) 111:1023–6. 10.1111/apa.16276 35098577 PMC9303202

[59] Larovere KL Riggs BJ Poussaint TY Young CC Newhams MM Maamari M Neurologic involvement in children and adolescents hospitalized in the United States for COVID-19 or multisystem inflammatory syndrome. *JAMA Neurol.* (2021) 78:536–47. 33666649 10.1001/jamaneurol.2021.0504 PMC7936352

[60] Ko Y Mendoza VM Mendoza R Seo Y Lee J Lee J Multi-faceted analysis of COVID-19 epidemic in the Republic of Korea considering Omicron variant: mathematical modeling-based study. *medRxiv.* [Preprint]. (2022). 10.1101/2022.04.15.22273907 PMC9259245 35790210

[61] He X Hong W Pan X Lu G Wei X. SARS-CoV-2 Omicron variant: characteristics and prevention. *MedComm.* (2021) 2:838–45. 10.1002/mco2.110 34957469 PMC8693031

[62] Yadav PD Sarkale P Razdan A Gupta N Nyayanit DA Sahay RR Isolation and characterization of SARS-CoV-2 Beta variant from UAE travelers. *J Infect Public Health.* (2022) 15:182–6. 10.1016/j.jiph.2021.12.011 34974274 PMC8704724

[63] Tegally H Wilkinson E Giovanetti M Iranzadeh A Fonseca V Giandhari J Detection of a SARS-CoV-2 variant of concern in South Africa. *Nature.* (2021) 592:438–43. 10.1038/s41586-021-03402-9 33690265

